# Retrieval-Augmented Generation-Enabled Multimodal Large Language Model for Histopathologic Grading of Cutaneous Squamous Cell Carcinoma and Melanocytic Nevi

**DOI:** 10.3390/cancers18152524

**Published:** 2026-08-06

**Authors:** Joshua Mijares, Eric Gan, Neil K. Jairath, Judy Hamad, Ahmed Alomari, Vignesh Ramachandran, Syril Keena T. Que

**Affiliations:** 1 Department of Dermatology, Indiana University School of Medicine, Indianapolis, IN 46202, USA; 2 Department of Pathology and Laboratory Medicine, Indiana University School of Medicine, Indianapolis, IN 46202, USA; 3 Prava Medical, San Francisco, CA 94105, USA

**Keywords:** cutaneous squamous cell carcinoma, melanocytic nevus, large language model, machine learning, artificial intelligence, histopathologic diagnosis, dermatopathology, retrieval-augmented generation

## Abstract

**Background**: Histopathologic grading is an essential step in guiding treatment recommendations and follow-up for cutaneous lesions. However, inter-observer variability in determining differentiation of cutaneous squamous cell carcinoma (cSCC) and dysplasia of melanocytic nevi remain a challenge. Retrieval-augmented generation (RAG)-assisted artificial intelligence may offer educational and diagnostic support in this process. **Methods**: Two isolated RAG pathways using Claude 4.5 Opus, each grounded via ChromaDB vector retrieval of task-specific literature, graded 60 cSCC cases by differentiation and 67 melanocytic nevus cases by dysplasia. Each case was analyzed three times, with the majority result used as the final grade. Concordance with reference dermatopathologist grading was calculated with 95% Wilson score confidence intervals; inter-rater reliability was assessed using Cohen’s kappa. **Results**: For cSCC, the LLM achieved 90.0% concordance (95% CI, 79.9–95.3%) with excellent agreement (κ = 0.85, 95% CI, 0.74–0.96). For nevi, concordance was 44.8% (95% CI, 33.5–56.6%) with agreement not statistically distinguishable from chance (κ = 0.072, 95% CI, −0.10–0.24). Discordant nevus classifications skewed toward the moderately dysplastic category (65.7% of LLM classifications vs. 41.8% of reference classifications), and no severely dysplastic case was concordantly classified (0/6). **Conclusions**: Despite an identical RAG architecture, concordance with reference dermatopathologist grading was markedly task-dependent: high for cSCC differentiation, low for nevus dysplasia grading. This pattern parallels reported human inter-rater reliability for these tasks, suggesting that task-specific validation is needed before clinical or educational use.

## Simple Summary

Diagnosing skin cancer and evaluating moles for signs of concern often relies on doctors examining tissue samples under a microscope, which is a process that can vary between experts. This study tested whether an artificial intelligence system, given access to relevant medical literature, could match a dermatopathologist’s grading of two types of skin lesions: squamous cell skin cancer and moles. The artificial intelligence agreed strongly with the dermatopathologist on skin cancer grading but showed almost no better than chance agreement on mole grading. These findings suggest artificial intelligence tools may already be useful for well-defined diagnostic tasks but need far more testing before being trusted for tasks where even human experts often disagree. This work highlights the importance of testing artificial intelligence separately for each diagnostic task rather than assuming one tool performs equally well everywhere.

## 1. Introduction

Cutaneous squamous cell carcinoma (cSCC) and dysplastic melanocytic nevi are two common entities in dermatopathology for which histopathologic grading directly determines management intensity. cSCC is the second most common skin cancer, with over one million new cases diagnosed annually in the United States alone, and its incidence continues to rise [1]. While the overall prognosis for cSCC is favorable, patients who do develop metastatic disease face substantially poorer outcomes [1]. The degree of differentiation has been shown to be a significant risk factor for metastasis and is incorporated into major staging and risk stratification systems, including AJCC8, the BWH staging system, and NCCN guidelines [2,3]. Poorly differentiated, very-high-risk cSCC warrants more aggressive treatment, including Mohs micrographic surgery and consideration of sentinel lymph node biopsy, while well- or moderately differentiated tumors without other high-risk features may be managed with electrodessication and curettage (ED&C) and less intensive surveillance [4]. Similarly, dysplastic nevi are morphologically and genomically distinct from common acquired nevi and are recognized as biomarkers of increased lifetime melanoma risk, though most melanomas arise de novo rather than from a preexisting nevus [5]. Nonetheless, the degree of dysplasia, based on architectural and cytological features, guides decisions on re-excision and surveillance intensity. For example, under the revised Melanocytic Pathology Assessment Tool and Hierarchy for Diagnosis (MPATH-Dx) classification schema, low-grade dysplastic nevi with positive margins (Class I) typically require no further treatment, whereas high-grade dysplastic nevi (Class V) are managed similarly to melanoma in situ, with re-excision generally indicated for positive margins [6,7].

However, despite the importance of grading in dermatopathology, inter-observer variability in the grading of both cSCC and melanocytic nevi is a well-recognized challenge. One study found inter-rater reliability for cSCC to be 0.56 [8]. Another study found that, for melanocytic nevi that were not diagnosed as melanoma in situ, the inter-rater agreement was 0.40 [9]. Because grading in both entities carries direct management consequences, tools capable of grading reliably and consistently may meaningfully impact clinical decision-making.

Artificial intelligence (AI) has emerged as a promising approach to augment histopathologic diagnosis in dermatopathology. Convolutional neural networks (CNNs) and other deep learning architectures have demonstrated high accuracy in classifying skin tumors on histopathologic images, including the differentiation of basal cell carcinoma, squamous cell carcinoma, melanoma, and nevi, with reported accuracies exceeding 90% in one study [10]. More recently, multimodal large language models (LLMs) have introduced the ability to interpret histopathologic images while simultaneously generating natural language explanations of their diagnostic reasoning [11]. This capacity for interpretable output distinguishes LLMs from traditional “black-box” deep learning classifiers and may be particularly valuable in educational settings, where trainees benefit from understanding the rationale behind a classification rather than receiving a label alone [12]. Furthermore, retrieval-augmented generation (RAG) has been shown to significantly enhance LLM performance in biomedical applications, with a meta-analysis demonstrating a pooled odds ratio of 1.35 (95% CI, 1.19–1.53) for improved performance compared to baseline LLMs [13]. RAG architectures ground model outputs in external, domain-specific knowledge by retrieving relevant documents from a curated knowledge base and incorporating them into the generation process, thereby reducing hallucinations and improving factual accuracy [14].

Despite these advances, many previous studies have only evaluated multimodal LLMs in the broad classification of the type of lesion [15,16,17]. To our knowledge, no prior study has evaluated the performance of RAG-enabled multimodal LLMs specifically for histopathologic grading tasks in dermatopathology. The purpose of this retrospective diagnostic concordance study was to evaluate whether a RAG-enabled multimodal LLM, using an identical underlying architecture, achieves comparable concordance with reference dermatopathologist grading across two distinct histopathologic grading tasks (cSCC differentiation and melanocytic nevus dysplasia) using Claude 4.5 Opus augmented with vector-based retrieval of expert consensus grading guidelines, across 127 total cases.

## 2. Materials and Methods

This retrospective diagnostic study evaluated the concordance of two RAG-enabled multimodal LLMs in grading cutaneous squamous cell carcinoma (cSCC) differentiation and melanocytic nevus dysplasia, respectively, with a reference dermatopathologist. The study was approved by the Indiana University Institutional Review Board (protocols #27844 and #27863).

We developed a dual-pathway computational system for grading dermatopathology images: one pathway was for the differentiation grade in cSCC, and one was for the dysplasia grade in melanocytic nevi. Each pathway combines a multimodal large language model with retrieval-augmented generation (RAG), so image interpretation is grounded in diagnostic criteria retrieved from the primary literature rather than the model’s internal knowledge alone. The two pathways remain fully isolated, with no shared context between tasks. For each pathway, the source literature was split into overlapping 1000-character chunks (200-character overlap) and embedded in a dedicated ChromaDB (version 1.0.12) collection using its default embedding function. The cSCC corpus comprised two articles [18,19]; the nevus corpus comprised four, spanning classical dysplastic nevus grading and the MPATH-Dx classification [7,20,21,22]. For each case, a criteria block was assembled by retrieving the five nearest chunks for each of a fixed set of subqueries (seven for cSCC, twelve for nevi) and concatenating the results.

**Figure 1:**
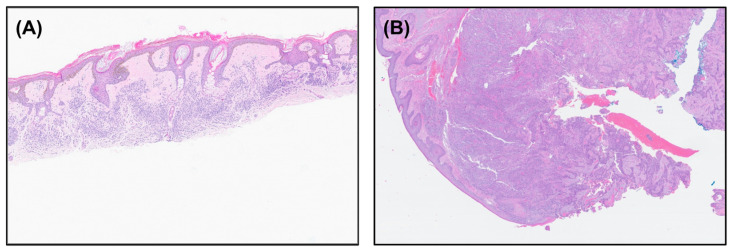
(**A**) Sample cSCC image used as input for LLM analysis. (**B**) Sample melanocytic nevus image used as input for LLM analysis.

Histopathologic images were obtained from the Indiana University Department of Pathology and PathPresenter. Cases were selected via consecutive sampling. Inclusion criteria required a confirmed histopathologic diagnosis of cSCC or melanocytic nevus and adequate tissue sampling. Cases were excluded if there was treatment prior to biopsy or insufficient image quality. A total of 60 cSCC reference cases (well-differentiated, n = 20; moderately differentiated, n = 22; poorly differentiated, n = 18 as graded by a reference dermatopathologist) and 67 melanocytic nevus reference cases (mild dysplasia, n = 33; moderate dysplasia, n = 28; severe dysplasia, n = 6 as graded by a reference dermatopathologist) were evaluated. Sample histopathologic images used in this study are included in Figure 1A,B. Reference grades were established by the same dermatopathologist across both cohorts, independently of and prior to LLM analysis, to avoid circularity between the reference standard and the model output under evaluation.

**Figure 2:**
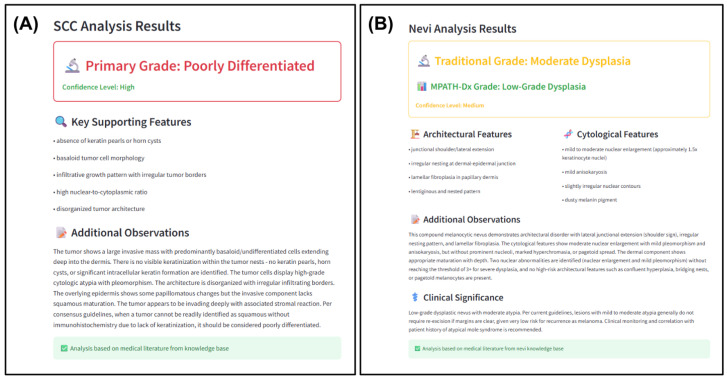
(**A**) Sample of the LLM’s automated interpretation of a cSCC after digital histopathologic image submission, including its decision-making logic and a narrative summary of the classification rationale. (**B**) Sample of the LLM’s automated interpretation of a melanocytic nevus after digital histopathologic image submission, including its decision-making logic and a narrative summary of the classification rationale.

Uploaded images were validated, size-constrained, and Base64-encoded. Both pathways used Anthropic’s claude-opus-4-5-20251101 (temperature 0.1; max output 1500 tokens for cSCC, 1000 for nevi), prompted in a single request combining the retrieved criteria, a task-specific instruction, and the image. Prompts framed the model as an expert dermatopathologist and specified explicit grading criteria and decision rules. For cSCCs, outputs were structured as a three-tier differentiation grade with supporting features. For nevi, outputs were structured as a three-tier dysplasia grade, a two-tier MPATH grade, a nuclear abnormality count, and feature lists for nevi. Structured parsing included rule-based fallbacks when needed. Sample model outputs are included in Figure 2A,B.

The system was built in Python (version 3.11.14) with Streamlit (version 1.45.1), LangChain (document ingestion/chunking; version 0.3.25), ChromaDB (vector storage/retrieval; version 1.0.12), and Pillow (image handling; version 11.2.1), with inference via the Anthropic API (version 0.52.2). The retrieval used ChromaDB’s default general-purpose embedding (all-MiniLM-L6-v2), rather than a domain-specific biomedical model; the embedding version was not pinned. The pipeline is reproducible given identical source documents, parameters, and settings, but the outputs are not bit-for-bit reproducible, since the underlying model is a fixed proprietary snapshot subject to future deprecation, and nonzero decoding temperature introduces run-to-run variation.

To reduce the influence of run-to-run output variability on the final grade, each case underwent repeat LLM analysis three times, with the final histologic grade determined by majority output. Overall and grade-specific concordance between LLM classification and the reference dermatopathologist’s grade were calculated as the proportion of matching classifications, with 95% confidence intervals computed using the Wilson score method. Inter-rater agreement was assessed using Cohen’s kappa coefficient, with 95% confidence intervals calculated as κ ± 1.96 × SE. All statistical analyses were performed using SPSS Version 31.0.

## 3. Results

Among the 60 cSCC cases evaluated, the RAG-enabled LLM demonstrated an overall concordance of 90.0% (95% CI, 79.9–95.3%) with the reference dermatopathologist’s differentiation grade (Table 1 and Table 2). Agreement was highest for well-differentiated tumors (100.0%, 95% CI, 83.9–100.0%; 20/20), followed by poorly differentiated tumors (88.9%, 95% CI, 67.2–96.9%; 16/18) and then moderately differentiated tumors (81.8%, 95% CI, 61.5–92.7%; 18/22). Cohen’s kappa was 0.85 (95% CI, 0.74–0.96), indicating excellent agreement by conventional benchmarks.

All six discordant cases involved disagreement between adjacent categories (well-versus-moderate or moderate-versus-poor). No case classified as well-differentiated by the reference dermatopathologist was assigned a poorly differentiated grade by the LLM, or vice versa (Table 1). Of the four discordant moderately differentiated reference cases, two were graded as well-differentiated and two as poorly differentiated by the LLM. Of the two discordant poorly differentiated reference cases, both were graded as moderately differentiated.

Among the 67 melanocytic nevus cases evaluated, the RAG-enabled LLM demonstrated an overall concordance of 44.8% (95% CI, 33.5–56.6%) with the reference dermatopathologist’s dysplasia grade (Table 3 and Table 4). Concordance was highest for moderately dysplastic nevi (64.3%, 95% CI, 45.8–79.3%; 18/28), followed by mildly dysplastic nevi (36.4%, 95% CI, 22.2–53.4%; 12/33). The LLM did not correctly classify any severely dysplastic nevus (0%, 95% CI, 0.0–39.0%; 0/6). Cohen’s kappa was 0.072 (95% CI, −0.10–0.24), indicating agreement not statistically distinguishable from chance.

Discordance followed a descriptive skew toward over-grading of dysplasia (Table 3). Of the 21 discordant mildly dysplastic reference cases, 20 were graded as moderately dysplastic and one as severely dysplastic by the LLM. All six severely dysplastic reference cases were graded as moderately dysplastic by the LLM. Among moderately dysplastic reference cases, discordant classifications were more often upgraded to severely dysplastic (8/28) than downgraded to mildly dysplastic (2/28). Overall, the LLM assigned a moderately dysplastic grade to 44 of the 67 cases (65.7%), compared with 28 of 67 (41.8%) by the reference dermatopathologist. However, given the low overall kappa, this pattern cannot be interpreted as a systematic bias.

To characterize retrieval behavior and illustrate the LLM’s reasoning process, we examined subquery-level retrieval yield for each pathway and reviewed representative case outputs. For cSCC, a seven-subquery panel retrieved 17 unique chunks from a 72-chunk corpus (23.6%). Representative subqueries included “well-differentiated squamous cell carcinoma characteristics keratinization” and “Broders grading system squamous cell carcinoma differentiation.” One retrieved passage indicated that keratin pearls, horn cysts, and intracellular keratin formation should each be weighed when assessing keratinization, with the least-differentiated area of the tumor guiding the final grade [19].

For nevi, the twelve-subquery panel retrieved 35 unique chunks from a 294-chunk corpus (11.9%). Representative subqueries included “mild dysplasia atypical melanocytic nevi characteristics” and “severe dysplasia atypical melanocytic nevi criteria.” One retrieved passage specified nuclear abnormality count thresholds distinguishing mild, moderate, and severe atypia [20]. Notably, in the nevus pathway, subqueries targeting mild, moderate, and severe dysplasia independently returned an overlapping set of the same four highest-ranked chunks, indicating that the general-purpose embedding function did not meaningfully distinguish query text differing only by grade-descriptor adjectives. This retrieval indistinctness is a plausible mechanistic contributor to the near-chance concordance observed for nevus grading.

Representative reasoning is illustrated for one discordant cSCC case and one concordant nevus case in Table 5. In the discordant cSCC case, the LLM graded a well-differentiated reference case as moderately differentiated. The model’s output correctly identified keratin pearl formation and intracellular keratinization but graded down based on moderate nuclear pleomorphism and infiltrative architecture despite recording mitotic activity as present but not prominent. The model’s stated rationale explicitly invoked a retrieved tie-breaking instruction to grade ambiguous or mixed tumors toward their least-differentiated component. In the concordant nevus case, both the LLM and reference dermatopathologist assigned severe dysplasia. The model’s rationale cited four nuclear abnormalities and multiple architectural high-risk features, which matched the retrieved threshold criteria for severe dysplasia.

## 4. Discussion

This study evaluated two RAG-enabled multimodal LLM pathways, one for cSCC differentiation grading and one for melanocytic nevus dysplasia grading, using an identical underlying model (Claude 4.5 Opus) and architecture, differing only in the retrieved literature and task-specific prompting. This design allowed us to isolate task from architecture: any difference in concordance could not be attributed to the RAG framework itself, but to how that framework performed relative to a reference standard across two distinct diagnostic tasks. To our knowledge, this is the first study to assess a RAG-augmented multimodal LLM specifically for histopathologic grading tasks in dermatopathology and the first to do so across two tasks in parallel using a shared architecture.

Concordance with the reference dermatopathologist differed substantially between tasks. For cSCC, the LLM demonstrated 90.0% concordance and excellent inter-rater agreement (κ = 0.85). For nevus dysplasia grading, concordance was 44.8%, with agreement not statistically distinguishable from chance (κ = 0.072, 95% CI, −0.10–0.24) and a directional pattern in discordant classifications toward the moderately dysplastic category (65.7% of LLM classifications vs. 41.8% of reference classifications). This pattern is consistent with published human inter-rater reliability for these same two tasks. Dermatopathologists and Mohs surgeons show only weak agreement on cSCC differentiation (κ = 0.56) [8], while agreement among pathologists on dysplastic nevus grading is comparably or more limited (κ = 0.40) [9]. These findings mirror the pattern of human–human agreement reported in the literature, where cSCC grading also shows higher inter-rater reliability than nevus dysplasia grading.

The divergence observed here may reflect the inherent reliability of the grading task itself, independent of whether the rater is human or AI, rather than a task-specific capability limitation of the LLM. Alternatively, given that subqueries corresponding to different nevus dysplasia grades returned an overlapping set of retrieved chunks, it may be possible that the general-purpose embedding model did not reliably distinguish grade-specific criteria language. This raises the possibility that part of the observed discordance reflects underspecified retrieval rather than an inherent limitation of the underlying multimodal model. Regardless, a kappa value indistinguishable from chance suggests that basic validity is unstable for nevus dysplasia grading, regardless of whether the second rater is a pathologist or an LLM. However, distinguishing between these possibilities is not possible with a single-rater design. It is possible that individual discordant cases reflect the reference dermatopathologist’s own diagnostic threshold rather than an objective error by the LLM. A multi-pathologist consensus panel, in which a majority or expert-adjudicated grade replaces the single-rater reference, would provide a more robust gold standard capable of distinguishing genuine model error from disagreement with one individual’s threshold.

Accurate and concordant grading carries direct management implications in both entities. Under the revised MPATH-Dx schema, low-grade dysplastic nevi with positive margins typically require no further treatment, while high-grade lesions warrant wide local excision [23]. Under NCCN guidelines, well-differentiated cSCC is managed less intensively than poorly differentiated disease, which warrants more aggressive margins [24]. Discordance in either direction risks either undertreatment or overtreatment, which is why task validation should precede any deployment.

In addition to their ability to provide diagnostic support, multimodal LLMs have a distinguishing feature compared to traditional convolutional neural network (CNN) classifiers in their capacity to generate natural language explanations of diagnostic reasoning, rather than functioning as “black-box” classifiers that provide a label without articulating the underlying features. This capacity may be most useful for educational purposes in tasks where concordance with expert grading is high, such as cSCC, where the LLM’s stated rationale could reinforce feature recognition for a learner, while tasks in grading nevus dysplasia, where concordance is lower, may result in confusion for the learner, regardless of which rater is “correct”.

Several limitations of this study warrant discussion. First, the use of selected image regions rather than whole slide images is not representative of routine clinical practice, where pathologists evaluate the entire tissue section. While this approach was necessary given the current input constraints of multimodal LLMs, it introduces a selection bias, as the diagnostic information available to the model was curated rather than comprehensive, which may inflate apparent concordance relative to what would be observed with unguided whole slide input and may limit immediate clinical applicability of these findings. Whole slide-level analysis has been shown to have improved model performance in precision and recall, even when using a tiled, multi-scale approach [25]. This is likely the next direction for deep learning models and eventually LLMs [26,27]. Second, the diagnoses were established by a reference dermatopathologist, but, given the inherent inter-observer variability in these grading tasks, even expert diagnoses carry a degree of uncertainty. This is a fundamental challenge in evaluating any diagnostic tool for tasks where the reference standard itself is imperfect. Third, the sample size of 127 cases, while sufficient for this initial proof-of-concept evaluation, limits the statistical power to detect differences in performance for the various grading subcategories. For example, in the severely dysplastic nevus subgroup (n = 6), wide confidence intervals preclude firm conclusions about LLM performance at this grade. Larger, multicenter test sets will be needed to adequately power subgroup-level comparisons in future work.

Future directions should include additional training and evaluation using larger, multi-institutional cohorts with diverse tissue preparation, staining, and scanning protocols, which would allow the RAG retrieval component to be tested for robustness across sites. It is also important to compare RAG-enabled LLM performance against both expert dermatopathologists and traditional CNN-based classifiers on the same case sets. Expansion to using whole slide images as input and assessment of the model’s performance across a wider spectrum of melanocytic lesions could make this tool more applicable and increase its utility in a clinical environment. Task-specific refinement of the RAG pipeline itself, such as via retrieval embeddings trained on dermatopathology-specific corpora rather than general-purpose embeddings, may also improve retrieval relevance for morphologically subtle tasks such as nevus dysplasia grading. Finally, the educational potential of RAG-enabled LLMs warrants formal evaluation, as the ability to generate structured, guideline-grounded explanations of grading decisions could serve as a valuable training tool for pathology residents and fellows.

## 5. Conclusions

This study demonstrates that concordance between a RAG-enabled multimodal LLM and reference dermatopathologist grading is highly task-dependent. This divergence parallels previously reported human inter-rater reliability for these same two tasks, suggesting that the observed differences may reflect the inherent reliability of each grading task rather than a fixed capability of the LLM itself. While early data may suggest feasibility in applying these grading tasks for cSCC, there may be challenges that remain before such tools can be integrated into standard clinical workflows for melanocytic nevi. Before RAG-enabled multimodal LLMs can be considered for clinical or educational use, each grading task will require independent validation, ideally against a multi-rater consensus reference standard capable of distinguishing true diagnostic accuracy from concordance with a single rater. Guideline-grounded reasoning and natural language explanations of such reasoning are meaningful advantages over CNNs with black-box approaches, but this explanatory capacity is only as trustworthy as the underlying concordance. If these limitations are addressed, then AI-assisted tools that improve standardization and reproducibility have the potential to enhance diagnostic consistency and clinical care.

## Figures and Tables

**Table 1:** Confusion matrices for cSCC grading, comparing RAG-enabled LLM classification output and reference dermatopathologist classification.

		LLM			
		Well	Moderate	Poor	Total
Reference	Well	**20**	0	0	20
	Moderate	2	**18**	2	22
	Poor	0	2	**16**	18
	Total	22	20	18	60

Note: Values represent number of cases. Diagonal elements (bolded) represent concordant classifications.

**Table 2:** Overall and grade-specific classification concordance and inter-rater reliability for cSCC grading between the RAG-enabled LLM and the reference dermatopathologist classification.

Metric	Value (95% CI)
Overall Concordance	90.0% (79.9–95.3%)
Concordance with Well-Differentiated Reference	100% (83.9–100.0%)
Concordance with Moderately Differentiated Reference	81.8% (61.5–92.7%)
Concordance with Poorly Differentiated Reference	88.9% (67.2–96.9%)
Cohen’s Kappa	0.85 (0.74–0.96)

**Table 3:** Confusion matrices for melanocytic nevus grading, comparing RAG-enabled LLM classification output and reference dermatopathologist classification.

		LLM			
		Mild	Moderate	Severe	Total
Reference	Mild	**12**	20	1	33
	Moderate	2	**18**	8	28
	Severe	0	6	**0**	6
	Total	14	44	9	67

Note: Values represent number of cases. Diagonal elements (bolded) represent concordant classifications.

**Table 4:** Overall and grade-specific classification concordance and inter-rater reliability for melanocytic nevus grading between the RAG-enabled LLM and the reference dermatopathologist classification.

Metric	Value (95% CI)
Overall Concordance	44.8% (33.5–56.6%)
Concordance with Mildly Dysplastic Reference	36.4% (22.2–53.4%)
Concordance with Moderately Dysplastic Reference	64.3% (45.8–79.3%)
Concordance with Severely Dysplastic Reference	0% (0.0–39.0%)
Cohen’s Kappa	0.072 (−0.10–0.24)

**Table 5:** Representative concordant and discordant case examples illustrating RAG-enabled LLM retrieval and reasoning for cSCC differentiation and melanocytic nevus dysplasia grading.

Pathway	Reference Grade	LLM Grade	Concordant	Key Retrieval Criteria Cited	Summary of Model Rationale
cSCC	Well-Differentiated	Moderately Differentiated	No	Keratin pearl formation; intracellular keratinization as qualifying features	Recorded keratinization consistent with well-differentiated criteria but graded down based on moderate pleomorphism and infiltrative architecture; invoked retrieved tie-breaking rule for ambiguous cases
Nevus	Severely Dysplastic	Severely Dysplastic	Yes	Nuclear abnormality count threshold (Pozo criteria) for severe atypia	Cited 4 nuclear abnormalities and multiple architectural high-risk features, meeting retrieved threshold for severe/high-grade dysplasia

## Data Availability

De-identified data may be made available from the corresponding author upon reasonable request, subject to institutional and IRB approval.

## Abbreviations

The following abbreviations are used in this manuscript:

AI	Artificial Intelligence
AJCC8	American Joint Committee on Cancer, 8th Edition
BWH	Brigham and Women’s Hospital
CI	Confidence Interval
CNN	Convolutional Neural Network
cSCC	Cutaneous Squamous Cell Carcinoma
ED&C	Electrodessication and Curettage
LLM	Large Language Model
MPATH-Dx	Melanocytic Pathology Assessment Tool and Hierarchy for Diagnosis
NCCN	National Comprehensive Cancer Network
RAG	Retrieval-Augmented Generation
SE	Standard Error
SPSS	Statistical Package for the Social Sciences
